# Effects of a Smartphone-Based Breastfeeding Coparenting Intervention Program on Breastfeeding-Related Outcomes in Couples During First Pregnancy: Randomized Controlled Trial

**DOI:** 10.2196/51566

**Published:** 2024-12-17

**Authors:** Yi-Yan Huang, Rong Wang, Wei-Peng Huang, Tian Wu, Shi-Yun Wang, Sharon R. Redding, Yan-Qiong Ouyang

**Affiliations:** 1 School of Nursing Wuhan University Wuhan China; 2 Department of Nursing Renmin Hospital of Wuhan University Wuhan China; 3 Department of Critical Care Medicine Sir Run Run Shaw Hospital Zhejiang University School of Medicine Hangzhou China; 4 Tongji Medical College, Huazhong University of Science and Technology Wuhan Central Hospital Department of Obstetrics Wuhan China; 5 Global Health of Project HOPE Washington, MD United States

**Keywords:** breastfeeding, co-parenting, randomized controlled, child, efficacy, depressive symptoms

## Abstract

**Background:**

A low breastfeeding rate causes an increased health care burden and negative health outcomes for individuals and society. Coparenting is an essential tactic for encouraging breastfeeding when raising a child. The efficacy of the coparenting interventions in enhancing breastfeeding-related outcomes is controversial.

**Objective:**

This study aimed to examine the effects of coparenting interventions on exclusive breastfeeding rates, exclusive breastfeeding duration, breastfeeding knowledge, parenting sense of competence, coparenting relationships, depressive symptoms in new couples at 1 and 6 months post partum, and the BMI of infants 42 days post partum.

**Methods:**

This was a randomized, single-blinded controlled clinical trial. Eligible couples in late pregnancy in a hospital in central China were randomly assigned to 2 groups. While couples in the control group received general care, couples in the intervention group had access to parenting classes, a fathers’ support group, and individual counseling. Data were collected at baseline (T0), 1 month post partum (T1), and 6 months post partum (T2). Data on exclusive breastfeeding rate and exclusive breastfeeding duration were analyzed using the chi-square, Fisher exact, or Mann-Whitney *U* tests; coparenting relationships and the infant’s BMI were analyzed using an independent samples *t* test; and breastfeeding knowledge, parenting sense of competence, and depressive symptoms were analyzed using a generalized estimation equation.

**Results:**

A total of 96 couples were recruited, and 79 couples completed the study. The intervention group exhibited significantly higher exclusive breastfeeding rates at T1 (90% vs 65%, *P*=.02) and T2 (43.6% vs 22.5%, *P*=.02), compared with the control group. Exclusive breastfeeding duration was extended in the intervention group than in the control group at T1 (30, range 30-30 days vs 30, range 26.5-30 days; *P*=.01) and T2 (108, range 60-180 days vs 89, range 28-149.3 days; *P*<.05). The intervention group exhibited greater improvements in maternal breastfeeding knowledge (β=.07, 95% CI 0.006-0.13; *P*=.03) and maternal parenting sense of competence (β=5.49, 95% CI 2.09-8.87; *P*<.01) at T1, enhanced coparenting relationships at T1 (*P*<.001) and T2 (*P*=.02), paternal breastfeeding knowledge at T2 (β=.25, 95% CI 0.15-0.35, *P*<.001), paternal parenting sense of competence at T1 (β=5.35, 95% CI 2.23-8.47, *P*<.01), and reduced paternal depressive symptoms at T2 (β=.25, 95% CI 0.15-0.35, *P*<.001), and there was a rise in infants’ BMI at 42 days post partum (β=.33, 95% CI 0.01-0.64, *P*=.04).

**Conclusions:**

An evidence-based breastfeeding coparenting intervention is effective in improving exclusive breastfeeding rate, prolonging exclusive breastfeeding duration within the initial 6 months post partum, enhancing parental breastfeeding knowledge, levels of parenting sense of competence and coparenting relationship, infant’s BMI, and reducing paternal depressive symptoms.

**Trial Registration:**

Chinese Clinical Trial Registry ChiCTR2300069648; https://tinyurl.com/2p8st2p8

## Introduction

Breastfeeding is a crucial public health issue that has shown health benefits for the mother and infant, providing optimal nutrition for infant growth, promoting immune substances that prevent respiratory infections, diarrhea, and otitis media [1,2]. Benefits to mothers include increased lactogen and oxytocin secretion, reduced risk of maternal diseases, and promoted emotional bonding with infants [3]. Exclusive breastfeeding (EBF) for infants up to 6 months of age, followed by continued breastfeeding for 2 years is recommended by the World Health Organization [4].

However, the current global breastfeeding landscape is bleak, with only 40% of infants under 6 months are exclusively breastfed across 194 nations. In China, the EBF rate stands at 34.1%, and in the United States, it is even lower at 25.8%, falling short of the World Health Organization’s target of 50% [5,6]. Low breastfeeding rates lead to increased health care costs and negative health outcomes. The cost of not breastfeeding in the United States was US $13 billion per year in direct medical costs and US $18 billion per year in indirect costs [7]. By enhancing breastfeeding practice, it is possible to prevent the death of 820,000 children each year, of which 87% are infants under 6 months of age [8].

The low breastfeeding rate is attributed to a lack of paternal involvement in childcare [9]. Cultural norms and traditional gender roles frequently discourage fathers from actively participating in breastfeeding. It is crucial to promote, support, and protect breastfeeding worldwide, as it embodies society’s shared responsibility [10]. There is an urgent demand for improved breastfeeding intervention programs that address the needs of both parents.

Bich et al [11] discovered that offering breastfeeding education and counseling services to fathers in the intervention group resulted in notably higher EBF rates at 4 and 6 months compared with the control group. Similarly, Su and Ouyang [12] illustrated that coparenting, accompanied by guidance on emotional and practical support, led to significantly increased rates of exclusive breastfeeding at 4 and 6 months, along with a reduction in infant formula usage. However, contrasting findings from other studies suggest that such interventions do not necessarily lead to improved EBF rates [13-15]. Therefore, more vigorously designed studies are required to fully comprehend the possible benefits and drawbacks of incorporating fathers into coparenting methods targeted at encouraging effective breastfeeding practices.

This randomized controlled study aimed to investigate the effects of a breastfeeding coparenting intervention program to breastfeeding-related outcomes in couples during their first pregnancy, including breastfeeding rate, breastfeeding duration, parental breastfeeding knowledge, parental parenting sense of competence, coparenting relationship, parental depressive symptoms, and infant BMI.

## Methods

### Study Design and Participants

This study was a randomized, single-blinded controlled clinical trial, including measurements of pretest at baseline (T0), posttest after intervention (1 month post partum) (T1), and at 6 months post partum (T2).

The inclusion criteria consisted of couples (1) in the first pregnancy ≥ 28 weeks, (2) planning to breastfeed their infant, and (3) able to complete the related questionnaire independently. Exclusion criteria were couples or one of the couples (1) having or had participated in previous breastfeeding-related research, (2) who are health care workers, and (3) unable to access the internet.

### Randomization and Blinding

Participants were couples recruited from an outpatient obstetrics department of a large hospital in central China using convenience sampling. Researchers provided a brief introduction of the study to the couples, who were then directed to a WeChat official account (a popular social platform). Informed consent was obtained, and participants were then given access to the account.

Eligible couples were randomly allocated into either the intervention group or the control group. A randomization sequence was generated by a random service. Participants couples in either the intervention or control group were strangers, therefore they were blinded to reduce contamination bias. Researchers who compiled and analyzed the research data didn’t take part in the intervention process. The outcome evaluators were blinded as they did not know the group assignments.

### Intervention

Based on the Breastfeeding Co-parenting Framework [16], a breastfeeding coparenting intervention program was developed through a literature review [17] and Delphi method. A total of 14 studies, conducted in 9 different countries between January 1995 and February 2022, were included. The coparenting breastfeeding interventions, including start and stop dates, duration, program components, and content were outlined. In total, 7 experts were invited to participate as consultants from April 21 to June 23, 2022. After 2 rounds of Delphi consultation involving 6 experts, the intervention program had good validity and reliability with a coefficient judgment basis of 0.93, a familiarity coefficient of 0.87, an authority coefficient of 0.90 and Kendall W of 0.62. The intervention program included a 7-session parenting course, a father’s support group and individual counseling from late pregnancy to 6 months post partum (Multimedia Appendix 1).

Couples in the control group received generally available care. Before childbirth, regular prenatal examinations were conducted in the hospital, during which time couples were guided on maternal nutrition, weight management, and breastfeeding. After delivery, regular home visits were performed by the community nurses, who assessed the growth and development of the baby, as well as the uterus recovery status, breastfeeding condition, and pelvic floor function and provided health education. Researchers contacted these female participants by phone each month.

In addition to the interventions administered to the control group, participants in the intervention group were requested to add the researchers to their personal WeChat accounts, join the WeChat intervention group, and engage with a WeChat public account named “Guardian of Maternal and Infant Health” (Multimedia Appendix 2). This account was developed by the research group.

The classes were provided from late pregnancy to 4 weeks post partum, covering a total of 7 topics with a new topic. Between 28 and 37 weeks of gestation, educational sessions focusing on breastfeeding significance and techniques were provided to the intervention group participants by WeChat video on the WeChat account. From 37 weeks of gestation until delivery, participants received information on breastfeeding in specific circumstances through textual and video materials delivered by the research team, supplemented by community nurses. In addition, on the 14th day post partum, researchers and community nurses engaged in face-to-face discussions with couples, offering guidance on infant growth characteristics and distributing informational leaflets. At 4 weeks post partum, screenings for postpartum depression were conducted during personal meetings with couples by researchers and community nurses, along with the provision of resources on postnatal mental well-being, including textual, visual, and video materials. On the 42nd day post partum, intervention participants and their newborns underwent health checkups conducted by community nurses. Researchers sent reminders to participants about these classes by WeChat or phone calls.

Besides, male participants in the intervention group were invited to join the WeChat father’s support group, providing them with a platform to freely express their feelings and concerns, which was not available to male participants in the control group. Individual counseling was also provided to the intervention group couples through direct meetings with content experts, WeChat calls or phone calls, when needed. In addition, couples could seek help through individual counseling (contact experts by WeChat or telephone call when needed). The intervention was conducted by the research team consisting of an international board-certified lactation consultant, a national second-level psychological counselor, and a postgraduate student major in maternal and child health.

### Outcomes

#### Primary Outcomes

The primary outcomes were EBF rate and duration. Exclusive breastfeeding is defined as feeding infants with breast milk only from birth, with no supplementation of any other fluids or food, except vitamin, mineral drops, or medication [18]. The EBF duration is the total time of EBF from initiation to cessation. Data on EBF rate and EBF duration were obtained by asking the question: “What is your current feeding mode (exclusive breastfeeding, mixed feeding, or exclusive formula feeding)?” and “How long have you been exclusively breastfeeding your infant (in days)?”

#### Secondary Outcomes

##### Breastfeeding Knowledge Questionnaire

Designed by Su and Ouyang [12], this 18-item questionnaire asks about the benefits and skills of breastfeeding. One point is awarded for a correct answer, for a total possible score of 18, with a higher score indicating more enhanced breastfeeding knowledge. The questionnaire had a high level of internal reliability (Cronbach **α**=0.82) [19].

##### Parenting Sense of Competence Scale

Originally developed by Johnston to assess parenting satisfaction and efficacy [20], Ngai et al [21] translated the scale into Chinese and validated it with good internal consistency (Cronbach α=0.85) and test-retest reliability (correlation coefficient=0.87). The scale consists of 17 items, each of which adopts a 6-point Likert scale, from “strongly disagree” to “strongly agree.” Items 2, 3, 4, 5, 8, 9, 12, 14, and 16 are inversely scored for a total possible score of the scale from 17 to 102 points. The higher the score, the higher the level of perceived competence of the parents.

##### Brief Co-Parenting Relationship Scale

Developed by Feinberg et al [22] in 2012, the Brief Co-parenting Relationship Scale measures the quality of coparenting relationships between parents when raising a child together. It is composed of 14 items and 7 subscales. The Chinese version was developed by Min Wu and Zhao [23], including 5 dimensions with 14 items. All items are scored on a 7-point Likert scale, with 3 items being reverse scored, for a total score of 0 to 84. A higher score indicates a better coparenting relationship. The split-half reliability coefficient for the total score was 0.516, and Cronbach α coefficient for the total score was 0.613.

##### Edinburgh Postnatal Depression Scale

Compiled by Cox and translated into Chinese by Lee et al [24], this scale measures postpartum depressive symptoms. There are 10 items, and each item is divided into 4 levels from never (0 points) to always (3 points) for a possible total score of 30 points. If the total score is greater than or equal to 9, the mother has a high risk of experiencing depression and should be referred to a health care provider for further evaluation. The Chinese version of the scale had good validity. The split-half reliability of the Edinburgh Postnatal Depression Scale (EPDS) was 0.74, Cronbach α was 0.78 and test-retest reliability was 0.90 [25].

##### BMI of Infant at 42 Days

BMI was calculated by weight and body length data extracted from the newborn’s community checkup.

Both female and male participants were required to complete the questionnaire at 3 time points. At T0, questionnaires were collected by trained researchers through a paper survey face to face in an outpatient of obstetrics department. A researcher-designed questionnaire was used for data collection, including sociodemographic characteristics of the participants, Breastfeeding Knowledge Questionnaire (BKQ), and EPDS. At T1, data on breastfeeding rates, breastfeeding duration, BKQ, Parenting Sense of Competence Scale (PSOC), EPDS, and Brief Co-Parenting Relationship Scale were collected in community visits through a paper survey. At T2, in addition to the data collected in T1, weight and body length data of the infants at 42 days post partum were obtained to calculate the BMI. At this time, data were collected through Wenjuanxing (an easy-to-use survey platform).

### Sample Size

Sample size was calculated based on the results of Bich and Cuong [26]. According to the calculation formula of PASS15.0, we calculated the sample size was 72 couples (power=0.80, α=.10). With a 20% attrition rate, the sample size of 90 participant couples was more adequate (total sample size). As a result, 90 participant couples were involved (45 couples in each group).

### Statistical Analysis

Statistical analysis was undertaken using SPSS (version 24.0; IBM Corp). The per-protocol approach was used to analyze data. Kolmogorov-Smirnov test was used for judgment of data normality. Continuous data are presented as median (IQR) or means (SD); classified data are presented as n (%). The Mann-Whitney *U* test, student *t* test, chi-square test, and the Fisher exact tests were used for data comparison at baseline. Data on EBF rates and EBF duration were analyzed using the chi-square test, Fisher exact tests, or Mann-Whitney *U* test. Data on coparenting relationship were analyzed by independent samples *t* test. To examine the influence of the intervention over time, data on breastfeeding knowledge, parenting sense of competence, coparenting relationship, and depressive symptoms were analyzed by a generalized estimation equation. The significance level is set at .05 (2-tailed).

### Ethical Considerations

The study was approved by the Ethics Committee of the Department of Medicine, Wuhan University (IRB2022015) and was registered at ChiCTR.org.cn (ChiCTR2300069648). We followed the CONSORT-EHEALTH (Consolidated Standards of Reporting Trials of Electronic and Mobile Health Applications and Online Telehealth) checklist (Multimedia Appendix 3) [27].

## Results

### Participant Characteristics

**Figure 1:**
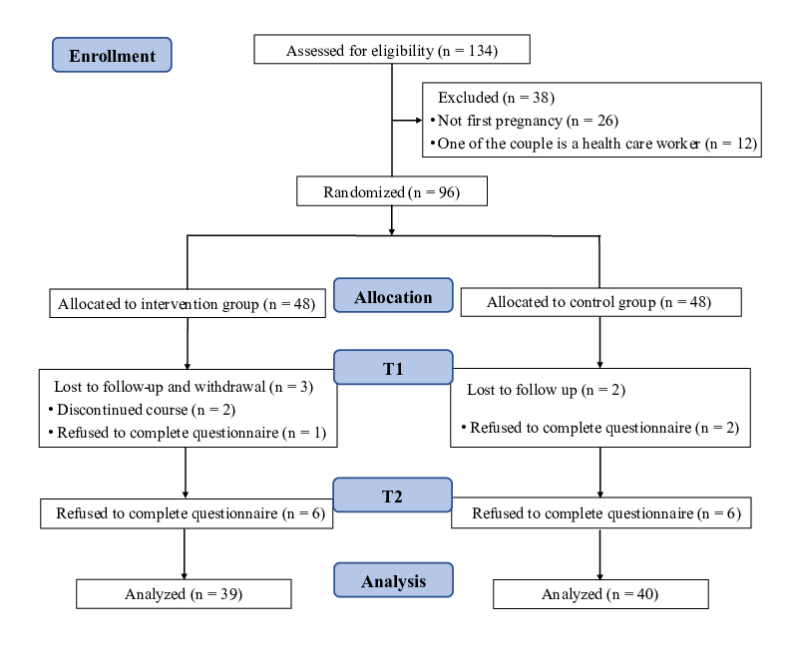
Flow diagram of participants’ inclusion process. T1: 1 month postpartum; T2: 6 months postpartum.

Sociodemographic characteristics of participants are presented in Table 1. From August 2022 to March 2023, 96 couples were recruited while 79 couples completed the research (39 couples in the intervention group, 40 couples in the control group). The attrition rate was 21.5% (17/79). A total of 17 participants dropped out because of discontinued courses or refusion to complete the survey (Figure 1).

**Table 1:** Baseline characteristics of participant couples (N=79).

Variables				Total (N=79)		Intervention group (n=39)		Control group (n=40)		*P* value
**Maternal**										
		Age (years), median (IQR)	30 (28-32)		30 (29-33)		30 (28-32)		.10	
		Urban residence, n (%)	76 (96.2)		38 (97.4)		38 (95)		>.99	
	**Educational level, n (%)**									.24
		High school or below	11 (13.9)		3 (7.7)		8 (20)		—^a^	
		Bachelor’s degree	60 (76)		31 (79.5)		29 (72.5)		—^a^	
		Postgraduate or above	8 (10.1)		5 (12.8)		3 (7.5)		—^a^	
	**Employment, n (%)**									.71
		Worker	1 (1.3)		1 (2.6)		0 (0)		—^a^	
		Administrator	33 (41.8)		17 (43.6)		16 (40)		—^a^	
		Teacher	8 (10.1)		5 (12.8)		3 (7.5)		—^a^	
		Private enterprise	25 (31.6)		12 (30.8)		13 (32.5)		—^a^	
		Free work	12 (15.2)		4 (10.3)		8 (20)		—^a^	
	**Average monthly household income (Yuan; 1 Yuan=US $0.14), n (%)**									.20
		≤3000	1 (1.3)		0 (0)		1 (2.5)		—^a^	
		3000-5000	12 (15.2)		3 (7.7)		9 (22.5)		—^a^	
		5001-10,000	34 (43)		18 (46.1)		16 (40)		—^a^	
		≥10,000	32 (40.5)		39 (49.4)		14 (35)		—^a^	
	**Maternal secondary outcomes at baseline, points**									
		BKQ^b^	0.70 (0.12)		0.71 (0.13)		0.69 (0.12)		.37	
		PSOC^c^	61.82 (5.70)		61.79 (5.84)		61.85 (5.63)		.97	
		EPDS^d^	8.0 (4-12)		8.0 (4-12)		7.5 (4-11)		.98	
**Paternal**										
	Age (years), median (IQR)		32 (30-35)		32 (30-35)		32 (30-34)		.64	
	**Educational level, n (%)**									.78
		High school or below	6 (7.6)		2 (5.1)		4 (10)		—^a^	
		Bachelor’s degree	63 (79.8)		32 (82.1)		31 (77.5)		—^a^	
		Postgraduate or above	10 (12.7)		5 (12.8)		5 (12.5)		—^a^	
	**Employment, n (%)**									.68^e^
		Worker	22 (27.9)		9 (23.1)		13 (32.5)		—^a^	
		Administrator	28 (35.4)		16 (41.0)		12 (30)		—^a^	
		Teacher	12 (15.2)		5 (12.8)		7 (17.5)		—^a^	
		Private enterprise	16 (20.3)		13 (33.3)		3 (7.5)		—^a^	
		Free work	1 (1.3)		1 (2.6)		0 (0)		—^a^	
	**Paternal secondary outcomes at baseline, points**									
		BKQ^b^	0.57 (0.13)		0.58 (0.14)		0.57 (0.12)		.77	
		PSOC^c^	60.27 (5.83)		60.72 (6.54)		59.83 (5.09)		.5	
		EPDS^d^	7.67 (3.28)		7.23 (3.89)		8.10 (2.53)		.24	
**Infant**										
	Gestational age (weeks), median (IQR)		39 (38-39)		38.5 (38-39)		39 (38-39)		.53	
	Delivery mode, vaginal, n (%)		38 (48.1)		21 (53.9)		17 (42.5)		.37	
	Infants' gender, female, n (%)		43 (54.4)		23 (59)		20 (50)		.5	
	Infants’ birth weight (kg), mean (SD)		3.16 (0.26)		3.18 (0.29)		3.15 (0.24)		.69	
	Infants’ birth length (cm), median (IQR)		50 (49-50)		50 (49-51)		50 (49-50)		.21	
	Infants’ BMI (kg/m^2^), mean (SD)		12.89 (1.3)		12.78 (1.29)		13.00 (1.32)		.46	

^a^—: not available.

^b^BKQ: Breastfeeding Knowledge Questionnaire.

^c^PSOC: Parenting Sense of Competence Scale.

^d^EPDS: Edinburgh Postnatal Depression Scale.

^e^Fisher exact test.

The median maternal age was 30 (IQR 28-32) years, and the median paternal age was 32 (IQR 30-35) years. The median gestational age of infants was 39 (IQR 38-39) weeks with 4 infants being premature. A total of 48.1% (38/79) of the infants were delivered vaginally, and 45.6% (36/79) of the infants were male. Among the maternal, paternal and infant data, no statistical differences existed regarding the baseline data (*P*>.05; Table 1).

### Primary Outcomes: Exclusive Breastfeeding Rate and Exclusive Breastfeeding Duration

Details of the primary outcomes are presented in Table 2. At T1, the intervention group exhibited a significantly higher EBF rate compared with the control group (89.7% vs 65%, *P*=.02). At T2, the EBF rate remained higher in the intervention group (43.6% vs 22.5%, *P*=.02). The intervention group had an extended EBF duration than the control group at T1 (*P*=.01) and T2 (*P*<.05).

**Table 2:** Effects of breastfeeding coparenting intervention program on breastfeeding rates and breastfeeding duration (N=79).

Time and variables			Total		Control group (N=40)		Intervention group (N=39)		*P* value
**T1^a^ Feeding mode, n (%)**									.02
	EBF^b^	61 (77.2)		26 (65)		35 (89.8)		—^c^	
	Mixed feeding	17 (21.5)		13 (32.5)		4 (10.2)		—^c^	
	Artificial feeding	1 (1.3)		1 (2.5)		0 (0)		—^c^	
	EBF duration (days)	30 (28-30)		30 (26.5-30)		30 (30-30)		.01	
**T2^d^ Feeding mode, n (%)**									.02
	EBF	26 (32.9)		9 (22.5)		17 (43.6)		—^c^	
	Mixed feeding	39 (49.4)		20 (50)		19 (48.7)		—^c^	
	Artificial feeding	14 (17.7)		11 (27.5)		3 (7.7)		—^c^	
	EBF duration (days)	102 (32-180)		89 (28-149.3)		108 (60-180)		.045	

^a^T1: 1 month post partum.

^b^EBF: exclusive breastfeeding.

^c^—: not available.

^d^T2: 6 months post partum.

### Maternal Secondary Outcomes

Secondary outcomes of couples are displayed in Table 3. The generalized estimation equation model revealed a significant interaction term concerning maternal BKQ correct rates (group×time) at T1 (β=0.07, 95% CI 0.006-0.13; *P*=.03), indicating that the intervention effectively improved maternal breastfeeding knowledge at T1. In addition, the model showed a significant interaction term concerning maternal PSOC scores (group×time) at T1 (β=5.48, 95% CI 2.09-8.87; *P*<.01), demonstrating that the intervention effectively improved maternal parenting sense of competence at T1.

**Table 3:** Effects of breastfeeding co-parenting intervention program on secondary outcomes (N=79).

Parental outcomes			Control group	Intervention group	Group effect			Time effect			Group*time effect	
					β (95% CI)	*P* value	β (95% CI)		*P* value	β (95% CI)		*P* value
**Maternal**												
	**BKQ^a^ correct rates, mean (SD)**		—^b^	—	0.03 (−0.03 to 0.08)	.03	—		—	—		—
		T0^c^	0.69 (0.12)	0.71 (0.13)	—	—	—		—	—		—
		T1^d^	0.68 (0.07)	0.77 (0.06)	—	—	0.01 (0.05 to 0.04)		.71	0.07 (0.006-0.13)		.03
		T2^e^	0.78 (0.09)	0.81 (0.12)	—	—	0.05 (0.01-0.09)		.01	−0.05 (−0.02 to 0.11)		.18
	**PSOC^f^, mean (SD)**		—	—	0.18 (−2.30 to 2.64)	.89	—		—	—		—
		T0^c^	61.85 (5.63)	61.79 (5.84)	—	—	—		—	—		—
		T1^d^	60.73 (4.83)	66.72 (3.55)	—	—	−1.13 (−3.56 to 1.31)		.37	5.48 (2.09-8.87)		<.01
		T2^e^	63.15 (4.53)	65.05 (6.44)	—	—	1.30 (−1.07 to 3.67)		.28	1.39 (−2.40 to 5.19)		.47
	**EPDS^g^, mean (SD)**		—	—	−0.40 (−2.85 to 2.06)	.75	—		—	—		—
		T0^b^	8.35 (5.99)	8.18 (5.15)	—	—	—		—	—		—
		T1^c^	5.65 (2.80)	2.67 (2.60)	—	—	−2.85 (−4.90 to −0.80)		<.01	−2.66 (−5.48 to 0.15)		.58
		T2^d^	7.85 (3.76)	6.69 (4.08)	—	—	−0.63 (−2.78 to 1.53)		.57	−0.86 (−3.90 to 2.17)		.06
**Paternal**												
	**BKQ^a^ correct rates, mean (SD)**		—	—	0.03 (−0.05 to 0.10)	.50	—		—	—		—
		T0^b^	0.54 (0.19)	0.58 (0.14)	—	—	—		—	—		—
		T1^c^	0.67 (0.12)	0.76 (0.13)	—	—	0.13 (0.07-0.19)		<.001	0.08 (−0.003 to 0.17)		.06
		T2^d^	0.63 (0.08)	0.80 (0.08)	—	—	−0.02 (−0.09 to 0.06)		.65	0.25 (0.15-0.35)		<.001
	**PSOC^f^, mean (SD)**		—	—	0.99 (−1.55 to 3.55)	.44	—		—	—		—
		T0^b^	59.83 (5.09)	60.82 (6.51)	—	—	—		—	—		—
		T1^c^	61.55 (5.09)	67.90 (4.04)	—	—	1.73 (−0.37 to 3.82)		.11	5.35 (2.23-8.47)		<.01
		T2^d^	60.10 (6.39)	63.85 (4.38)	—	—	0.28 (−2.15 to 2.70)		.82	2.75 (−0.96 to 6.46)		.15
	**EPDS^g^, mean (SD)**		—	—	0.03 (−0.05 to 0.10)	.50	—		—	—		—
		T0^b^	9.82 (3.86)	8.21 (5.82)	—	—	—		—	—		—
		T1^c^	4.90 (2.38)	2.00 (0.95)	—	—	0.13 (0.07-0.19)		<.001	0.08 (−0.003 to 0.17)		.06
		T2^d^	11.75 (2.95)	5.03 (2.99)	—	—	−0.02 (−0.09 to 0.06)		.65	0.25 (0.15-0.35)		<.001
**Infant**												
	**Weight, mean (SD)**		—	—	0.07 (−0.06 to 0.20)	.29	1.07 (0.10-1.14)		<.001	0.09 (−0.05 to 0.24)		.21
		At birth	3.15 (0.24)	3.18 (0.29)	—	—	—		—	—		—
		42 days post partum	4.17 (0.40)	4.29 (0.42)	—	—	—		—	—		—
	**Body length, median (IQR)**		—	—	0.62 (−0.10 to 1.35)	.09	5.06 (4.64-5.49)		<.001	0.10 (−0.75 to 0.95)		.81
		At birth	50 (49-50)	50 (49-51)	—	—	—		—	—		—
		42 days post partum	54.25 (53-56)	55 (53-56)	—	—	—		—	—		—
	**BMI, mean (SD)**		—	—	−0.22 (−0.78 to 0.35)	.46	—		—	—		—
		At birth	14.20 (1.31)	12.78 (1.29)	—	—	—		—	—		—
		42 days post partum	14.17 (1.33)	13.00 (1.32)	—	—	1.18 (1.01-1.34)		<.001	0.33 (0.01-0.64)		.04

^a^BKQ: Breastfeeding knowledge questionnaire.

^b^Not available.

^c^T0: at baseline.

^d^T1: 1 month post partum.

^e^T2: 6 months post partum.

^f^PSOC: Parenting Sense of Competence Scale.

^g^EPDS: Edinburgh Postnatal Depression Scale.

**Figure 2:**
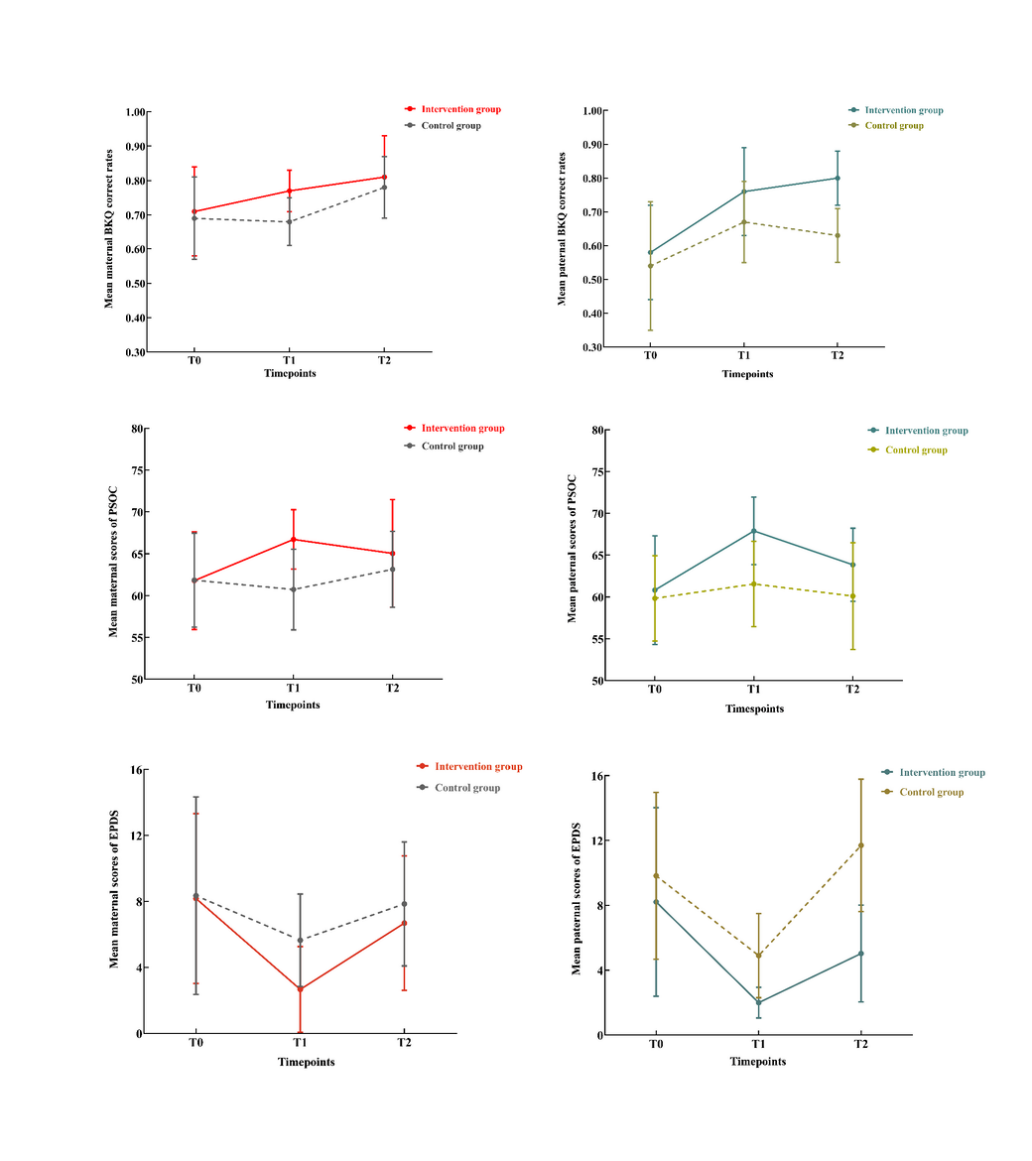
Comparison of the changes in secondary outcomes between groups. EPDS: Edinburgh Postnatal Depression Scale; PSOC: Parenting Sense of Competence Scale; T0: at baseline; T1: 1 month post partum; T2: 6 months post partum.

Since coparenting relationships were measured only twice, independent-sample *t* tests were conducted to compare the data. The intervention group exhibited superior coparenting relationships compared with the control group at both T1 (53.67, SD 5.80 vs 47.63, SD 6.21; *P*<.001) and T2 (53.46, SD 6.28 vs 49.70, SD 7.32; *P*=.02). Also, a significant reduction in depressive symptoms was observed at T1 (*P*< .01). To provide a clearer understanding of the study findings, Figure 2 illustrates the changes in secondary outcomes between the groups.

### Paternal Secondary Outcomes

The generalized estimation equation model showed significant interaction term in relation to paternal BKQ correct rates (group×time) at T2 (β=0.25, 95% CI 0.15-0.35; *P*<.001), verifying the intervention program’s efficacy in enhancing paternal breastfeeding knowledge at T2. Similarly, the model displayed significant interaction terms concerning paternal PSOC scores (group×time) at T1 (β=5.35, 95% CI 2.23-8.47; *P*<.01), affirming the intervention program’s effectiveness in improving paternal parenting sense of competence at T1. In addition, there were significant interaction terms observed regarding paternal EPDS scores (group×time) at T2 (β=0.25, 95% CI 0.15-0.35; *P*<.001), validating the intervention program’s success in reducing paternal depressive symptoms at T2. Furthermore, the model identified significant interaction terms regarding infants’ BMI (group×time; β=0.33, 95% CI 0.01-0.64; *P*=.04), confirming the intervention program’s positive impact on improving infants’ BMI at 42 days post partum.

## Discussion

### Principal Results

The main finding of this study was that the breastfeeding coparenting intervention program could effectively improve EBF rates. This is consistent with the study Bich et al [11], who conducted an intervention that provided breastfeeding education materials, counseling services at commune health centers, and household visits to fathers, resulting in a significant increase in exclusive breastfeeding rates at 4 and 6 months. Active participation of fathers in breastfeeding education and support sessions can enhance their understanding of breastfeeding’s importance, fostering a supportive home environment [28,29]. Fathers’ emotional, practical, and physical support is crucial for successful breastfeeding, easing the mothers’ burden and promoting coparenting collaboration [30].

Although higher than the 40% reported by Su and Ouyang [12], EBF rates fell short of the 55.56% reported by Abbass-Dick et al [14]. This discrepancy may be due to Canada’s higher breastfeeding prevalence rate compared with China. Nevertheless, the rates still did not meet the World Health Organization’s target of 50%. This study highlights the critical role of spousal support in promoting breastfeeding practices. Breastfeeding is a social responsibility that involves individuals, families, and society as a whole. In addition, workplace breastfeeding support and public mother-and-baby rooms are also essential factors in driving change [3,31]. Further work is needed to address these systemic factors. At the same time, the intervention program successfully extended EBF duration by providing comprehensive education and support, enabling participants to overcome breastfeeding-related challenges and maintain EBF for a long period, which is crucial for infant growth and development [3]. The findings highlight the importance of providing adequate support and resources to new parents to promote breastfeeding practices.

This study laid a firm foundation for enhancing breastfeeding knowledge throughout the first 6 months post partum, which is congruent with the findings of Bich and Cuong [26]. After the breastfeeding intervention program, maternal and paternal breastfeeding knowledge had improved significantly and experienced a slight decline over time, albeit the total improvement was maintained. Initially, paternal breastfeeding knowledge was substantially lower than maternal knowledge, but the gap was narrowed following the intervention, which encouraged both parents to participate and become more engaged in breastfeeding. By the end of the intervention, the knowledge of both mothers and fathers had improved significantly and tended to decline over time, although knowledge levels remained high.

The intervention effectively enhanced the levels of parenting sense of competence in both parents, which is similar to the findings of Chu et al [32], who reported that participants who received a mobile phone intervention were more likely to feel confident in adopting effective parenting skills and demonstrated a higher level of parenting sense of competence. Greater learned resourcefulness and social support were directly related to maternal role competence and pleasure [33]. High levels of learned resourcefulness enable parents to effectively deal with obstacles. Highly resourceful parents might use learned resourcefulness abilities like problem-solving tactics to reduce or eliminate troubling thoughts and sensations. Consequently, they are more effective, experience less emotional disturbance, and feel more capable and content when faced with the challenges of new parenthood.

The intervention had no significant effect on alleviating maternal depressive symptoms. The sample of parents reported low scores at baseline on the EPDS, providing a limited possibility for decreasing. In addition, it is also possible that the amount of information conveyed by the intervention was insufficient to elicit a significant reduction in depressive symptoms. Professional therapies, including interpersonal counseling and cognitive behavioral therapy provided by a trained psychotherapist, are necessary for effectively treating perinatal depression [34,35]. In this study, fathers in the control group were at risk of experiencing depressive symptoms at 6 months post partum, which was consistent with the findings of Cameron et al [36]. However, this intervention proved to be effective in alleviating paternal depression at T2. The most common help-seeking barrier reported by parents can refer to a lack of information when needed. The study approach provided content involving mental health and individual counseling, which offered some information support to parents.

This intervention had a notable impact on enhancing coparenting relationships, which differs from the findings of Abbass-Dick et al [13]. The enhancement observed in coparenting relationships may be related to the lessening of conflict. In this study, the researchers included components in which parents jointly developed breastfeeding objectives, accepted responsibility, provided feeding assistance, and developed strong communication and problem-solving skills, thereby minimizing parental conflicts.

Previous studies have examined the effects of breastfeeding coparenting interventions on perception of infants using a 5-point scale [37] and infantile development status using Developmental Milestones Checklist-II [38]. This is the first study to examine the impact of coparenting interventions on an infant’s BMI. To collect data on infants of participants in this study during the follow-up period, the researchers referred to health records established in the community. Typically, mothers take their newborns to for wellness checkups at 42 days following delivery. However, data collected at only 42 days post partum are insufficient for determining long-term changes. Therefore, it is necessary to monitor the growth and development of newborns for longer periods.

In total, 17 couples dropped out, although strategies were adopted to reduce the number of participants who dropped out, including reminding participants to engage in the study and gathering feedback from participants through telephone calls. More effort is needed to successfully engage parents in the intervention programs, such as offering tangible rewards or benefits to participants to complete the intervention successfully.

The coparenting intervention program for breastfeeding in this study was comprehensive and scientifically informed, drawing on a literature review and expert consultation. The study used both e-health technologies and face-to-face interactions to provide health information. As one of the few randomized controlled studies examining the impact of a breastfeeding coparenting intervention program on breastfeeding-related outcomes in couples having a first pregnancy, the results suggest that delivering health interventions using a video presentation and chatroom format may be an effective approach to improving outcomes.

### Strengths and Limitations

Dedicated to providing a holistic approach that addressed various aspects of postpartum well-being for both mothers and fathers, this study used a comprehensive intervention approach that included spouses as participants. Study outcomes were assessed 6 months post partum using a range of indicators. The interventions were designed based on theoretical frameworks and implemented under the guidance of multidisciplinary experts.

Nevertheless, this study is subject to several limitations. First, the relatively short follow-up period of 6 months post partum may not fully capture the enduring effects of the interventions on breastfeeding practices. Extending the follow-up duration to 2 years or more in future investigations would provide a more comprehensive understanding of the maintained impact on breastfeeding-related outcomes over time. Second, the sample predominantly comprised participants from urban areas with access to extensive medical services, potentially limiting the generalizability of the findings. This demographic skew potentially constrains the generalizability of the findings to populations with differing socioeconomic backgrounds or geographic locations. To enhance external validity, future studies should strive to recruit a more diverse sample, including couples from rural or remote areas. Finally, while the study effectively evaluated outcome measures, it notably lacked an assessment of process evaluation aspects, such as parental satisfaction with the intervention strategies used. Integrating process evaluation components into future research initiatives would not only provide valuable insights into the acceptability and feasibility of intervention approaches but also facilitate iterative improvements and refinements to enhance program effectiveness.

### Conclusions

This evidence-based breastfeeding coparenting intervention effectively enhanced EBF rates and prolonged breastfeeding duration at 1 and 6 months post partum. It also improved maternal and paternal breastfeeding knowledge, parenting sense of competence, coparenting relationship, alleviated paternal depression, and increased infants’ BMI at 42 days post partum. Further research is required to evaluate the long-term effects of the intervention across diverse geographic locations and integrate both outcome and process evaluations for a comprehensive understanding of its effectiveness and implementation.

## Appendix

Multimedia Appendix 1 Structure of the breastfeeding co-parenting intervention program.

Multimedia Appendix 2 Screenshots of the WeChat account.

Multimedia Appendix 3 CONSORT-EHEALTH (Consolidated Standards of Reporting Trials of Electronic and Mobile Health Applications and Online Telehealth) checklist.

## Abbreviations

BKQ: Breastfeeding Knowledge Questionnaire
CONSORT-EHEALTH: Consolidated Standards of Reporting Trials of Electronic and Mobile Health Applications and Online Telehealth
EBF: Exclusive breastfeeding.
EPDS: Edinburgh Postnatal Depression Scale.
PSOC: Parenting Sense of Competence Scale.
