# Supplementary material for: Effects of a Smartphone-Based Breastfeeding Coparenting Intervention Program on Breastfeeding-Related Outcomes in Couples During First Pregnancy: Randomized Controlled Trial
Source: J Med Internet Res. 2024 Dec 17;26:e51566. doi: 10.2196/51566 (PMC11688581; doi:10.2196/51566)
Supplement: Multimedia Appendix 1 [file jmir_v26i1e51566_app1.docx]

**Multimedia Appendix 2.** Structure of the breastfeeding co-parenting intervention program.

| Occurrence | Content | Format | Components |
| --- | --- | --- | --- |
| 28 weeks of pregnancy | Welcome, informed consent, completion of socio-demographic questionnaire | Face-to-face at prenatal examination |  |
| 30 weeks of pregnancy | Why breastfeed?  1. Benefits of breastfeeding 2. Disadvantages of artificial feeding | Online education  WeChat video |  |
| 32 weeks of pregnancy | How to breastfeed? 1. Proper time to breastfeed 2. Correct breastfeeding position 3. Judgment of starvation and satiety  4. Breast care during breastfeeding  5. Nutrition needs of breastfeeding mothers | Online education  WeChat video | Joint breastfeeding goal setting |
| 34 weeks of pregnancy | Preparation for childbirth: essential items for newborns | Face-to-face, booklet |  |
| 36 weeks of pregnancy | Breastfeeding under special cases: breast tenderness, chapped nipples, mastitis, insufficient breast milk | Online education  Tencent Conference |  |
| After birth | 1. Breast milk jaundice  2. Diarrhea of infant  3. Breastfeeding in the COVID-19 era  4. Breastfeeding when mother-infant separation  5. Breastfeeding while taking medication | Face-to-face  Postpartum unit | Shared breastfeeding responsibility |
| Two weeks postpartum | Characteristics of newborn growth  1. Sleep pattern of newborn  2. Routine newborn care (bathing, skin care, touch)  3. Infant vaccines and schedule 4. Common newborn illnesses | Face to face  During community visits | Parent-child interaction |
| Four weeks postpartum | 1. Postpartum recovery of mothers  2. Mental health of breastfeeding women 3. Parents’ mental health status prior to childbirth 4. Parents’ mental health status after childbirth   (One week before delivery) 5. Healthy diet after childbirth  (One week before delivery) | Face-to-face  During community visits | Proactive breastfeeding support |
| 1~6 months postpartum | 1. Fathers’ support group: chatroom communication  2. Individual counseling | Face-to-face or  Online when needed | Productive communication and problem-solving skills |
