# Supplementary material for: Effects of a Smartphone-Based Breastfeeding Coparenting Intervention Program on Breastfeeding-Related Outcomes in Couples During First Pregnancy: Randomized Controlled Trial
Source: J Med Internet Res. 2024 Dec 17;26:e51566. doi: 10.2196/51566 (PMC11688581; doi:10.2196/51566)
Supplement: Multimedia Appendix 2 [file jmir_v26i1e51566_app2.pdf]

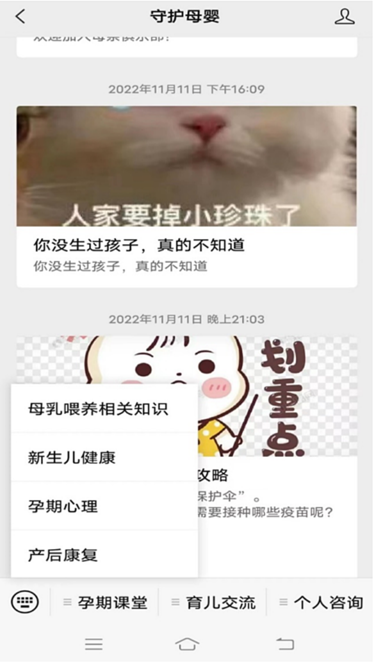
守护母婴
2022年11月11日 下午16:09
人家要掉小珍珠了
你没生过孩子，真的不知道
你没生过孩子，真的不知道
2022年11月11日 晚上21:03
划重点
母乳喂养相关知识
新生儿健康
孕期心理
产后康复
攻略
保护伞”。
需要接种哪些疫苗呢？
孕期课堂
育儿交流
个人咨询

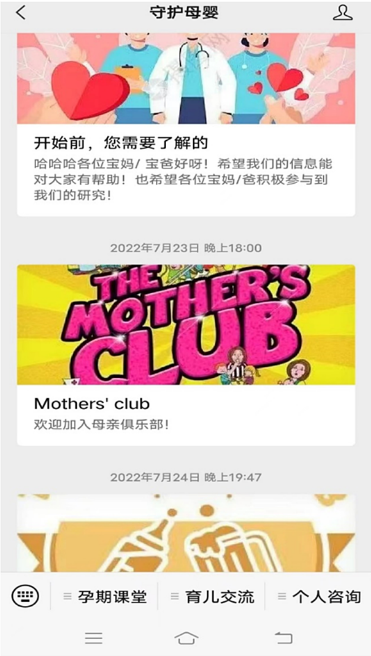
守护母婴
开始前，您需要了解的
哈哈哈各位宝妈/宝爸好呀！希望我们的信息能对大家有帮助！也希望各位宝妈/爸积极参与到我们的研究！
2022年7月23日 晚上18:00
THE MOTHER'S CLUB
Mothers' club
欢迎加入母亲俱乐部！
2022年7月24日 晚上19:47
孕期课堂
育儿交流
个人咨询
